# Long-term metabolic effects of malnutrition: Liver steatosis and insulin resistance following early-life protein restriction

**DOI:** 10.1371/journal.pone.0199916

**Published:** 2018-07-02

**Authors:** Prasad S. Dalvi, Steven Yang, Nathan Swain, Junsoo Kim, Senjuti Saha, Celine Bourdon, Ling Zhang, Rose Chami, Robert H. J. Bandsma

**Affiliations:** 1 Translational Medicine Research Program, The Hospital for Sick Children, Toronto, ON, Canada; 2 Morosky College of Health Professions and Sciences, Gannon University, Erie, PA, United States of America; 3 Department of Laboratory Medicine and Pathology, Faculty of Medicine, University of Toronto, Toronto, ON, Canada; 4 Department of Pediatric Laboratory Medicine, Hospital for Sick Children, Toronto, ON, Canada; 5 Department of Nutritional Sciences, Faculty of Medicine, University of Toronto, Toronto, ON, Canada; Universidade do Estado do Rio de Janeiro, BRAZIL

## Abstract

Early postnatal-life malnutrition remains prevalent globally, and about 45% of all child deaths are linked to malnutrition. It is not clear whether survivors of childhood malnutrition suffer from long-term metabolic effects, especially when they are later in life exposed to a fat and carbohydrate rich obesogenic diet. The lack of knowledge around this dietary “double burden” warrants studies to understand the long-term consequences of children previously exposed to malnutrition. We hypothesized that an early-life nutritional insult of low protein consumption in mice would lead to long-term metabolic disturbances that would exacerbate the development of diet-induced insulin resistance and non-alcoholic fatty liver disease (NAFLD). We investigated the effects of feeding a low protein diet (4% wt/wt) immediately after weaning for four weeks and subsequent feeding of a high carbohydrate high fat feeding for 16 weeks on metabolic function and development of NAFLD. Mice exposed to early-life protein restriction demonstrated a transient glucose intolerance upon recovery by regular chow diet feeding. However, protein restriction after weaning in mice did not exacerbate an obesogenic diet-induced insulin resistance or progression to NAFLD. These data suggest that transient protein restriction in early-life does not exacerbate an obesogenic diet-induced NAFLD and insulin resistance.

## Introduction

Despite all global efforts, malnutrition is still a major burden in developing countries and is considered the most relevant risk factor for illness and death of millions of young children. In the latest report in 2013, 45% of all child deaths were attributed to undernutrition, which translated to 3.1 million deaths worldwide [1]. Stunting and wasting affected at least 165 million and 52 million children, respectively [1]. Severe malnutrition is acutely associated with profound and ill-understood metabolic disturbances, such as, hepatic steatosis, and abnormal glucose homeostasis [2–6].

The increase in obesity and associated chronic metabolic disorders is a major public health problem not only in developed nations but also in low-income and middle-income countries [1]. The prevalence of overweight and obesity has increased by at least 4.9% between 1980 and 2013 in children and adolescents in developing countries [7]. However, obesity increasingly coexists with malnutrition in developing countries [1]. Extensive epidemiological studies have indicated that malnutrition during intrauterine and perinatal life has deleterious health effects that can last throughout the life of an individual [8–10]. This process, termed nutritional or developmental programming, allows specific physiological and metabolic adaptations that persist even after the early nutritional stress is resolved [11]. This programming is mediated by epigenetic alterations that change long-term gene expression and result in maintenance of the acquired phenotype [12–14].

Severe acute malnutrition in children and animal models has been specifically found to be associated with impaired glucose clearance related to pancreatic β-cell dysfunction [2, 15, 16]. Using an early-life protein restriction animal model, our group has showed that malnutrition leads to hepatic steatosis associated with severe mitochondrial dysfunction in young rats, underscoring the severity of malnutrition-induced metabolic disturbances [17, 18]. Yet, the long-term metabolic effects of early childhood malnutrition are not well known [19]. However, with the changes in socioeconomic status and dietary trends occurring in many developing countries, those who survive childhood malnutrition could be prone to becoming overweight and be more susceptible to chronic metabolic disorders in later-life [1], such as type 2 diabetes and non-alcoholic fatty liver disease (NAFLD) [20]. In recent years, NAFLD has become a common cause of chronic liver disease in obese individuals [20]. NAFLD ranges from simple hepatic steatosis to the more severe non-alcoholic steatohepatitis (NASH) with hepatic fibrosis, and frank cirrhosis [21]. In the current project, we sought to establish how early-life protein deficiency followed by exposure to an obesogenic diet exacerbates metabolic disturbances in mice, in particular, with regards to NAFLD and dysregulation of glucose homeostasis.

## Methods

### Experimental animals and diets

**Fig 1:**
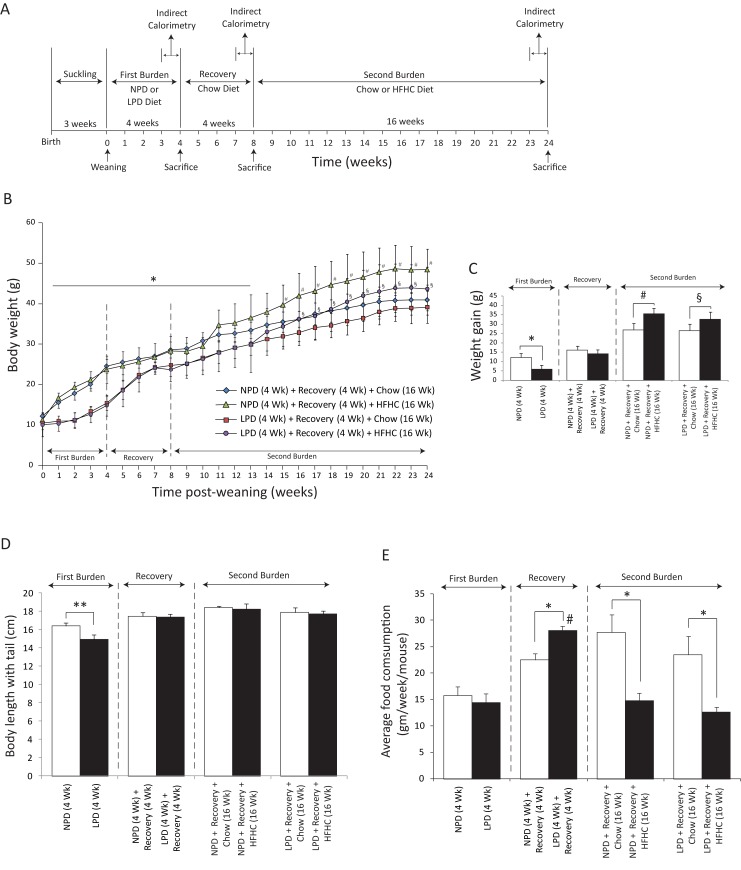
Assessment of weight gain, growth, stunting, pancreas weight and liver weight in C57BL/6 mice fed a low protein diet (LPD, malnutrition diet) or isocaloric normal protein diet (NPD, control diet) followed by a recovery period of chow diet feeding for 4 weeks and final exposure to a chow or HFHC diet for 16 weeks. (A) Experimental scheme: Mice were given each diet post weaning. The first four weeks post weaning are designated as the period of the first burden/malnutrition, the next 4 weeks of chow diet feeding are the period of recovery and the last 16 weeks are the period of second burden. For indirect calorimetric analysis, the mice were placed in metabolic cages 3 times, each time approximately one week prior to the beginning of a new diet regimen or before sacrifice. (B) Body weight change was assessed weekly over the entire experimental period of 24 weeks. Weight of NPD-fed mice was significantly higher than the LPD-fed mice starting at week 1 through week 13 (All data are presented as means ± SD, n ≥ 4 mice per group; two-way ANOVA; interaction *P < 0.05, Tukey’s *post hoc* test). The NPD/HFHC-fed mice weighed significantly higher than the NPD/chow-fed mice (All data are presented as means ± SD, n ≥ 4 mice per group, #< 0.05, Tukey’s *post hoc* test). Similarly, the LPD/HFHC-fed mice weighed significantly higher than the LPD/chow-fed mice (All data are presented as means ± SD, n ≥ 4 mice per group, §< 0.05, Tukey’s *post hoc* test). (C) At the end of each feeding period, the total amount of weight gained from the time of weaning was calculated. NPD-fed mice gained more weight that the LPD-fed mice at the end of the first 4 weeks of feeding (All data are presented as means ± SD, *P < 0.05, Tukey’s *post hoc* test). There was no statistical difference in the weight gain between NPD- or LPD-fed mice at the end of recovery period of 4 weeks. The NPD- or LPD-fed mice followed by recovery and HFHC feeding for 16 weeks gained significantly more weight than the NPD- or LPD-fed mice followed by recovery and chow diet feeding (All data are presented as means ± SD, n ≥ 4 mice per group, # and §< 0.05, Tukey’s *post hoc* test). (D) Final lengths of entire body with tail were measured at the end of each feeding period. These lengths were smaller in the LPD-fed mice than the NPD-fed mice at the end of the first 4 weeks of feeding (n ≥ 4 mice per group, **P< 0.01, Student's *t-*test). (E) Average weekly food intake was measured during each feeding period. There was no significant difference in the weekly food intake among the LPD- and NPD-fed mice during the first burden/malnutrition period. During the recovery period, the weekly food intake in the LPD-fed mice had significantly increased as compared with the NPD-fed mice. During the period of HFHC diet feeding both the NPD- and LPD-fed mice had significantly decreased weekly food intake (All data are presented as means ± SD, n ≥ 4 mice per group, *P < 0.05, ^#^*P* < 0.05 vs. LPD-fed mice, Tukey’s *post hoc* test, One-way ANOVA).

All studies were approved by the Institutional Animal Care and Use Committee at the Hospital for Sick Children, Toronto, Canada, in accordance with the Animal Welfare Act guidelines. Wild type C57BL/6 mice were purchased from Charles River Laboratories (Montreal, PQ, Canada) and mated. We used 13 pregnant mice in the study, and each litter had an average of 3–4 males and 3–4 females. After weaning the male mice, we randomized mice from each litter to either a low (4%) protein diet (LPD, first burden and period of malnutrition) or a normal (18%) protein diet (NPD) groups. All mice were housed in micro-isolator cages and maintained on a 12-h light (7:00 A.M.)/dark (7:00 P.M.) cycle with *ad libitum* access to rodent chow and water. At weaning, male pups were randomly exposed to 4 dietary regimens: 4 weeks of either a low (4%) protein diet (LPD, first burden and period of malnutrition) or a normal (18%) protein diet (NPD) (Harlan, Madison, WI, USA), followed by 4 weeks of recovery for all mice. Hereafter, mice were fed either a regular chow diet or high-fat high-carbohydrate diet (HFHC, second burden) for 16 weeks (Fig 1A). Each group had minimum 4 mice (n ≥ 4 mice per group). All intervention diets were provided *ad libitum*. LPD and NPD diets were isocaloric and matched in micronutrient content, except the proteins in the LPD were replaced by starch (Table 1). The HFHC diet was composed of a high-fat diet with 58% kcal from fat (D12331 from Research Diets, New Brunswick, NJ) (Table 1), and deionized distilled water enriched with a high fructose corn syrup equivalent that provided a total of 42 g/L of carbohydrates at a ratio of 55% of fructose (Acros Organics, Morris Plains, NJ) and 45% of sucrose by weight (Sigma-Aldrich, St. Louis, MO). This fat- and carbohydrate-rich obesogenic diet has been shown to induce liver steatosis and inflammation [22]. Mice were closely monitored for signs of illness, and the body weight and food intake were measured weekly. Mice were sacrificed at 3:00 pm after a 6 h fast with a CO_2_ chamber. Tail and body lengths were measured, liver and pancreas were excised and weighed, and the liver was either snap-frozen in liquid nitrogen and stored at -80°C or processed for histology. During tissue and organ harvesting, we dissected out pancreas very carefully using dissecting microscope in order to avoid either losing any pancreatic tissue or collecting surrounding connective tissue. We adopted the similar strategy for harvesting the livers as well.

**Table 1:** Composition of diets.

Ingredient	Regular Chow Diet		Normal-Protein Diet (NPD)		Low-Protein Diet (LPD)		High-Fat Diet (HFD)	
	% weight	% kcal	% weight	% kcal	% weight	% kcal	% weight	% kcal
**Protein**	20.3	20.8	18.3	19.6	4.1	4.4	23.0	16.4
**Carbohydrate**	66.0	67.7	62.8	67.2	75.4	82.2	35.5	25.5
**Fat**	5.0	11.5	5.5	13.2	5.5	13.4	35.8	58.0
**Kcal/g**	3.85		3.7		3.7		5.56	

### Characterization of the mouse model of NAFLD/NASH

Histology: Tissues were fixed in 10% neutral buffered formalin solution (Sigma, St. Louis, MO) for 48 h and paraffin-embedded. Sections were cut at 5 μm and stained with hematoxylin and eosin to evaluate steatosis, inflammation, hepatocellular injury, or stained with Masson's trichrome (Trichrome Stain—Masson Kit, Sigma-HT15) to evaluate fibrosis by light microscopy.

Evaluation of steatosis by image analysis: Computer-based image analyses were carried out using the software Image Pro Plus, version 10.0 for Windows (Media Cybernetics, Rockville, MD, USA). Digital images were prepared for the analysis as follows: (a) hepatic tissue images were segmented using the manual function, (b) the threshold tool was used to differentiate fat vesicles from the rest of the liver tissue, (c) the percent area occupied by fat vesicles (steatosis) in the whole image was determined by using the software’s count tool, (d) the fat vesicles were automatically detected and quantified by the software. This method was performed with paraffin -embedded liver samples stained with hematoxylin and eosin and was based on the previously published methods [23, 24].

Indirect calorimetry: To study energy expenditure, we used metabolic cages from Columbus Instruments, Columbus, OH (Oxymax Lab Animal Monitoring System: CLAMS). Animals were individually housed for 48 hours with free access to water and their assigned diets. Each animal was placed 3 times in the metabolic chambers: 1) after 3 weeks of LPD or NPD, 2) after 3 weeks of recovery, and 3) after 15 weeks of either HFHC or NPD (Fig 1A). The first 24 h were used for acclimatization, and analysis was conducted on data collected from hours 25 to 48. The respiratory exchange ratio (RER) was calculated as the ratio of carbon dioxide production (VCO_2_) to oxygen consumption (VO_2_). Values were normalized to body weight. Metabolic cages comply with the guidelines stipulated in the U.S. Department of Health and Human Services “Guide for the Care and Use of Laboratory Animals”.

### Glucose tolerance test and insulin resistance

Glucose was measured at 2:00 pm at the end of each dietary period following a 5 h fast. For intraperitoneal glucose tolerance tests (IPGTT), blood samples were drawn from the tail vein at 0, 10, 20, 30, 60, 90, and 120 minutes after i.p. injection of 2 g/kg dextrose. Blood glucose was determined using a One Touch II glucose monitor (LifeScan Inc., Milpitas, CA, USA). Fasting insulin was measured in plasma obtained from 50–100 μl of blood collected from tail vein before glucose injection (time 0). Blood was collected in microvettes (Sarstedt, Montreal, Québec, Canada), and plasma separated by centrifugation at 4°C (2000g X 10 min) and stored at -80°C until assayed. Insulin was measured in plasma using a mouse ELISA kit (Linco Research, St. Charles, MO), and using mouse insulin as a standard (Crystal Chem Inc.). Insulin resistance was calculated using Homeostasis Model Assessment of insulin resistance (HOMA-IR) [25].

### Hepatic triglyceride quantification

Liver triglyceride levels were determined as previously described [26]. Briefly, 100 mg of wet liver tissues was homogenized and an enzymatic assay was performed using Triglyceride Quantification Assay kit (Colorimetric/Fluorometric) purchased from Abcam (ab65336). Photometric absorbances were read at 570 nm.

### Quantitative PCR

RNA was isolated from liver using purelink RNA mini columns (Ambion, Life Technologies, Carlsbad, CA). A total of 1 μg of RNA was used to synthesize cDNA with the high capacity cDNA archive kit (ABI, Applied Biosystems, Life Technologies, Carlsbad, CA). Quantitative PCR for the genes of profibrogenesis, proinflammatory cytokines, inflammasome component, markers of inflammatory cells and oxidative stress, and ketohexokinase was conducted with specific primers designed using Integrated DNA Technologies PrimerQuest online software [27] (Table 2), and platinum SYBR green qPCR supermix-UDG w/ROX per manufacturer’s instructions. cDNA samples (25 ng) and dose curve samples (range of 1 ng to 100 ng) were loaded in triplicate for each gene. Samples were run on a 384-well block ABI 7900HT fast real-time PCR system and analyzed using SDS 2.4 software (ABI, Applied Biosystems, Life Technologies, Carlsbad, CA). Reverse transcriptase negative controls were run for each primer set. The following cycling conditions were used: 50°C for 2 min, 95°C for 2 min, and 40 repeats of 95°C for 15 sec to 60°C for 1 min, followed by melting curve analysis at 95°C for 15 sec to 60°C for 15 sec then 95°C for 15 sec.

**Table 2:** List of gene-specific SYBR primers.

Gene	Forward	Reverse
**Housekeeping gene**		
18S	5’-AGTCCCTGCCCTTTGTACACA-3’	5’-CGATCCGAGGGCCTCACTA-3’
**Fibrogenic genes**		
Collagen α1(I)	5’-TAGGCCATTGTGTATGCAGC-3’	5’-ACATGTTCAGCTTTGTGGACC-3’
Collagen α1(IV)	5’-CACATTTTCCACAGCCAGAG-3’	5’-GTCTGGCTTCTGCTGCTCTT-3’
PAI-1	5’-GCCAGGGTTGCACTAAACAT-3’	5’-GCCTCCTCATCCTGCCTAA-3’
TGFβ1	5’-GTGGAAATCAACGGGATCAG-3’	5’-ACTTCCAACCCAGGTCCTTC-3’
TIMP1	5’-AGGTGGTCTCGTTGATTTCT-3’	5’-GTAAGGCCTGTAGCTGTGCC-3’
α-SMA	5’-AAACAGGAATACGACGAAG-3’	5’-CAGGAATGATTTGGAAAGGA-3’
**Proinflammatory cytokines**		
TNFα	5’-AGGGTCTGGGCCATAGAACT-3’	5’-CCACCACGCTCTTCTGTCTAC-3’
IL-1α	5’-CCAGAAGAAAATGAGGTCGG-3’	5’-AGCGCTCAAGGAGAAGACC-3’
IL-1β	5’-GGTCAAAGGTTTGGAAGCAG-3’	5’-TGTGAAATGCCACCTTTTGA-3’
MCP-1	5’-ATTGGGATCATCTTGCTGGT-3’	5’-CCTGCTGTTCACAGTTGCC-3’
**Inflammasome component**		
NLRP3	5’-AAGTAAGGCCGGAATTCACC-3’	5’-AAAATGCCTTGGGAGACTCA-3’
**Marker of inflammation (infiltration of inflammatory cells)**		
CD68	5’-ACCGCCATGTAGTCCAGGTA-3’	5’-ATCCCCACCTGTCTCTCTCA-3’
F4/80	5’-CATAAGCTGGGCAAGTGGTA-3’	5’-GGATGTACAGATGGGGGATG-3’
**Markers of oxidative stress**		
CYP2E1	5’-AGGCTGTCAAGGAGGTGCTAC-3’	5’-GTTTCCCCATTCCCCAGTC-3’
Nrf2	5’-TCTTCCATTTACGGAGACCCA-3’	5’-GATTCACGCATAGGAGCACTG-3’
Nqo1	5’-TCAACTGGTTTACAGCATTGGC-3’	5’-GCTTGGAGCAAAATAGAGTGGG-3’
Gstp1	5’-ACCCTGCTGTCCCAGAACC-3’	5’-GCGAGCCACATAGGCAGAG-3’
**Ketohexokinase (KHK) primers**		
KHK-A	5’-TTGCCGATTTTGTCCTGGAT-3’	5’-CCTCGGTCTGAAGGACCACAT-3’
KHK-C	5’-TGGCAGAGCCAGGGAGAT-3’	5’-ATCTGGCAGGTTCGTGTCGTA-3’

### Statistical analysis

Results are presented as either frequency (%) or as mean ± SD or mean ± SEM as appropriate. Statistical comparisons were performed using two-tailed Student’s *t*-test, one-way or two-way ANOVA with the *post hoc* Tukey’s test. The statistical analysis was performed with assistance from GraphPad Prism Software Version 6.00 (GraphPad Software, San Diego California USA). For all statistical analyses, *P*<0.05 was considered significant.

## Results

### Impact of a nutritional double burden on weight gain and stunting

We first characterized the impact on weight gain and stunting in C57BL/6 mice of a LPD feeding followed by 4 weeks of recovery with regular chow diet and then by HFHC diet feeding for 16 weeks (Fig 1A). During the first burden (i.e. week 1–4), compared with the mice fed the isocaloric NPD, the mice fed LPD gained significantly less weight (weight in grams, NPD versus LPD, 12.36 ± 0.99 versus 5.98 ± 0.79; *P* < 0.05) and were stunted (length in cm, NPD versus LPD, 16.40 ± 0.21 versus 14.95 ± 0.25; *P* < 0.01) (Fig 1B–1D). During recovery with regular chow diet feeding for 4 weeks (i.e. week 5–8), the malnourished mice demonstrated catch-up growth and gained about 8.26 g compared to 3.81 g in animals fed a NPD (weight in grams, NPD versus LPD, 16.17 ± 1.05 versus 14.24 ± 0.78; *P* < 0.05) (Fig 1C). However, this catch up growth was not sufficient for malnourished animals to attain the body weight of animals fed the isocaloric NPD (Fig 1B and 1C). The stunting induced by LPD feeding was no longer evident after total 20 weeks of chow diet feeding (Fig 1D). Measurements of average weekly food intake during each feeding period indicate that there was no significant difference in the weekly food intake among the LPD and NPD-fed mice during the first burden/malnutrition period, however, during the recovery period, the weekly food intake in the LPD-fed mice had significantly increased as compared with the NPD-fed mice (Fig 1E). During the 16 weeks of obesogenic (second) burden, the two animal groups fed HFHC diet became significantly more obese compared to the mice kept on regular chow during this period (Fig 1B and 1C). During this period of HFHC diet feeding both the NPD- and LPD-fed mice had significantly decreased weekly food intake (Fig 1E).

### Impact of a nutritional double burden on whole body metabolism

**Fig 2:**
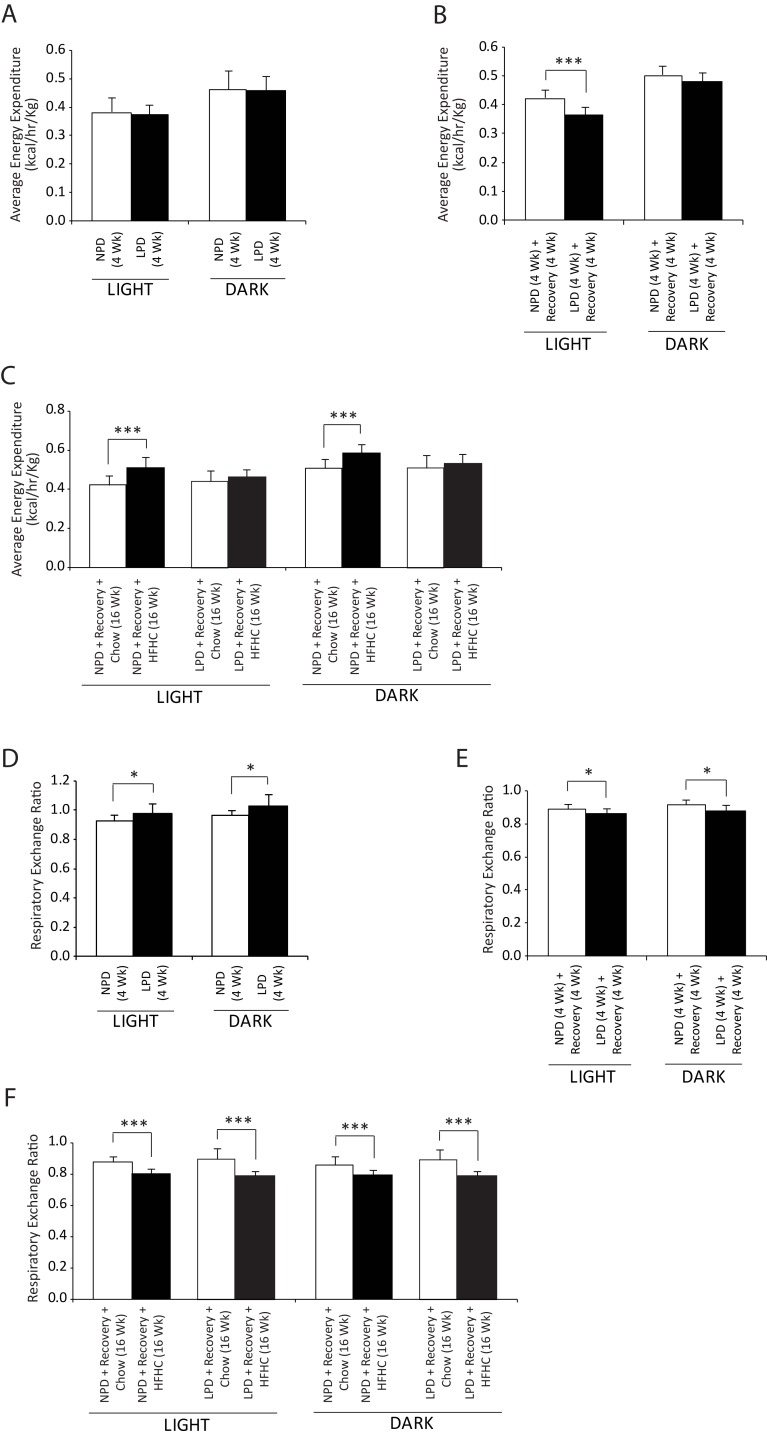
Assessment of whole body metabolism in resting condition in C57BL/6 mice at the end of 4 weeks of normal protein diet (NPD, control diet) or low protein diet (LPD, malnutrition diet) feeding during the first burden/malnutrition period, 4 weeks of chow diet feeding during the recovery period, and 16 weeks of chow or HFHC diet feeding during the second burden period. (A-C) Energy expenditure, and (D-F) respiratory exchange ratio in the resting condition over a 24-hour monitoring period were determined by indirect calorimetry as described in the methods section (All data are presented as means ± SD, n ≥ 4 mice per group, *P < 0.05, ***P < 0.001, Tukey’s *post hoc* test, One-way ANOVA).

We subjected C57BL/6 mice to indirect calorimetry at the end of each feeding period. We calculated metabolic rate on the basis of energy expenditure and determinations of O_2_ consumption and CO_2_ production in resting mice (Fig 2A and 2C). During the initial period of exposure to the LPD diet, mice did not show any difference in energy expenditure compare to NPD-fed mice (Fig 2A), whereas at the end of recovery period, the LPD-fed mice demonstrated decreased energy expenditure during the light phase (Fig 2B). At the end of HFHC diet feeding, an increase in energy expenditure was found compared to the chow-fed control mice during both the light and dark phases in the NPD-fed group, whereas the LPD-fed mice did not show any difference (Fig 2C). Thus, these findings indicate that energy expenditure in the LPD-fed or NPD-fed mice was not affected by the protein content in the diet during the initial feeding period, however, a significant decrease in energy expenditure was found in the LPD-fed mice during the recovery period indicating energy conservation in these mice. The RER of the LPD-fed mice was significantly higher than in NPD-fed mice during the light phase (NPD versus LPD, 0.93 ± 0.01 versus 0.98 ± 0.01; *P* < 0.05) as well as the dark phase (NPD versus LPD, 0.97 ± 0.02 versus 1.03 ± 0.02; *P* < 0.05) (Fig 2D). After 4 weeks of nutritional recovery, the RER in the LPD-fed mice was found to be lower during the light phase (ratio, NPD versus LPD, 0.89 ± 0.01 versus 0.86 ± 0.01; *P* < 0.05) and the dark phase (ratio, NPD versus LPD, 0.92 ± 0.01 versus 0.88 ± 0.01; *P* < 0.05). The RER was lower in all animals fed HFHC diet, with no difference between early-life NPD or LPD feeding (Fig 2F). Overall, the changes in the RER indicate that the LPD-fed mice utilize carbohydrates as the main energy source during the period of LPD feeding, whereas during the recovery and HFHC diet feeding period, these mice utilize fat as the main energy fuel.

### Impact of nutritional double burden on glucose regulation

**Fig 3:**
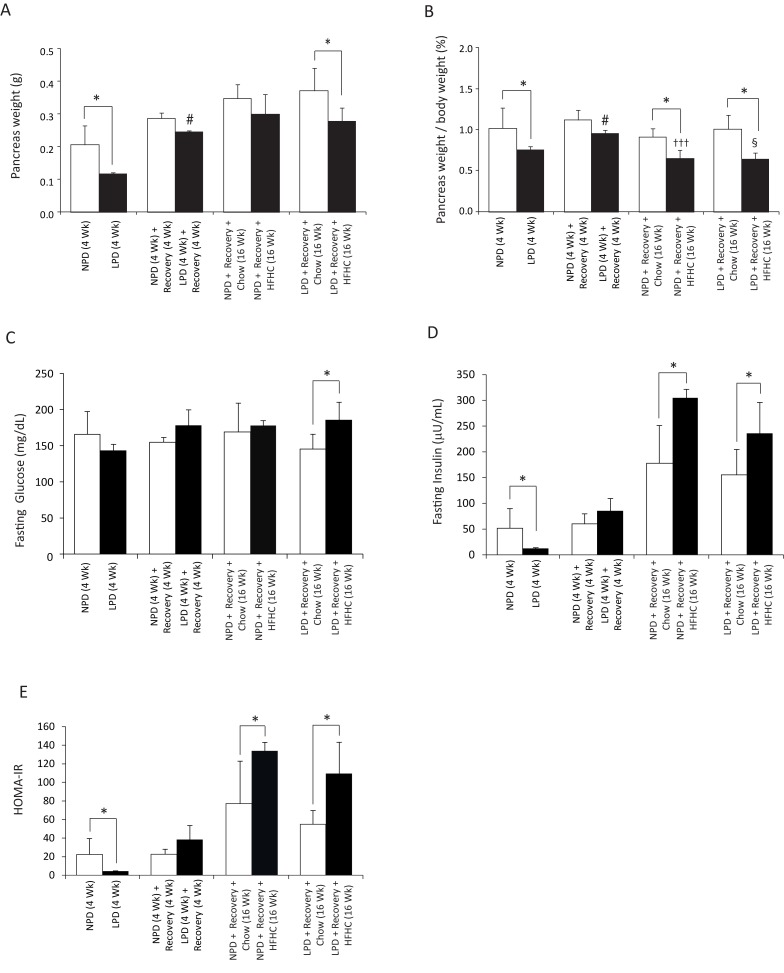
Assessment of pancreas weight, fasting glucose and insulin, and insulin resistance in C57BL/6 mice at the end of 4 weeks of normal protein diet (NPD, control diet) or low protein diet (LPD, malnutrition diet) feeding during the first burden/malnutrition period, 4 weeks of chow diet feeding during the recovery period, and 16 weeks of chow or HFHC diet feeding during the second burden period. (A) Mouse pancreata were weighed *ex vivo* after each feeding period. Only the LPD-fed mice, and the LPD/HFHC-fed mice had lower absolute pancreas weights in comparison to the matching control mice (All data are presented as means ± SD, n ≥ 4 mice per group; *P < 0.05, ^#^*P* < 0.05 vs. LPD-fed mice at the end of 4 weeks of first burden/malnutrition period, Tukey’s *post hoc* test, One-way ANOVA). (B) Mouse pancreata were weighed *ex vivo* after each feeding period. The LPD-fed mice at the end of 4 weeks of first burden/malnutrition period and 16 weeks of HFHC diet feeding had lower pancreas-to-body weight ratios in comparison to the matching control mice. At recovery, the pancreas-to-body weight ratio were significantly increased for the LPD-fed mice as compared with the first burden/malnutrition period. The NPD-fed mice had lower pancreas-to-body weight ratio at the end of16 weeks of HFHC diet feeding (All data are presented as means ± SD, n ≥ 4 mice per group; *P < 0.05, ^†††^*P* < 0.001 vs. NPD-fed mice following recovery, ^§^*P* < 0.05 vs. LPD-fed mice following recovery, ^#^*P* < 0.05 vs. LPD-fed mice at the end of 4 weeks of malnutrition period, Tukey’s *post hoc* test, One-way ANOVA). (C) Fasting blood glucose levels were measured at necropsy. The fasting blood glucose level was significantly higher only in the LPD/HFHC-fed mice at the end of 16 weeks of HFHC feeding (All data are presented as means ± SD, n ≥ 4 mice per group, *P < 0.05, Tukey’s *post hoc* test, One-way ANOVA). (D) Fasting insulin levels were measured at necropsy. Only the LPD-fed mice had lower fasting insulin levels in comparison to the NPD-fed control mice at the end of malnutrition period, and the NPD/HFHC- and LPD/HFHC-fed mice had higher fasting insulin levels than the corresponding chow diet-fed control groups (All data are presented as means ± SD, n ≥ 4 mice per group, *P < 0.05, Tukey’s *post hoc* test, One-way ANOVA). (E) Homeostasis model assessment of insulin resistance (HOMA-IR) was calculated using the data from fasting plasma glucose and insulin levels. Only the LPD-fed mice had lower HOMA-IR in comparison to the NPD-fed control mice, and the NPD/HFHC- and LPD/HFHC-fed mice had higher HOMA-IR than the corresponding chow diet-fed control groups. There was no statistical difference in the HOMA-IR between NPD-fed and LPD-fed mice following recovery period (All data are presented as means ± SD, n ≥ 4 mice per group, *P < 0.05, Tukey’s *post hoc* test, One-way ANOVA).

**Fig 4:**
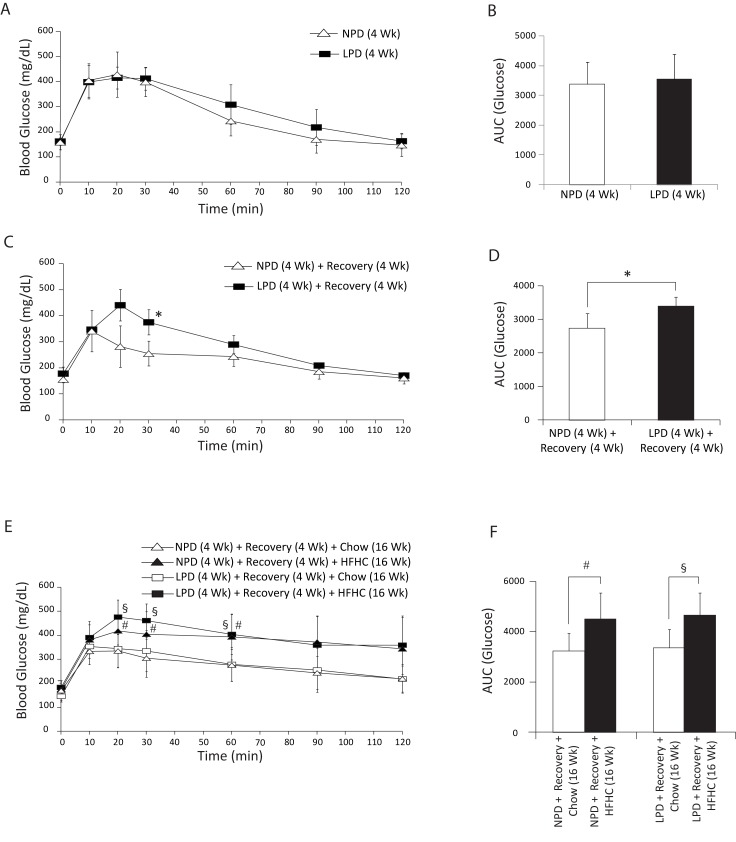
Assessment of glucose tolerance in C57BL/6 mice at the end of 4 weeks of normal protein diet (NPD, control diet) or low protein diet (LPD, malnutrition diet) feeding during the first burden/malnutrition period, 4 weeks of chow diet feeding during the recovery period, and 16 weeks of chow or HFHC diet feeding during the second burden period. (A, C, E) All intraperitoneal glucose tolerance tests (IPGTT) were performed during 2 h after glucose injection in mice that were fasted for 6 h. (B, D, F) AUC for the IPGTTs was calculated from 0 to 120 min. The LPD-fed mice following recovery and also LPD/HFHC mice demonstrated increased AUC (glucose) relative to the matched control mice. The NPD/HFHC-fed mice also had higher increased AUC relative to matched control mice. There was no statistical difference in the AUC between NPD-fed and LPD-fed mice at the end of 4 weeks of malnutrition period (All data are presented as means ± SD, n ≥ 4 mice per group, *P < 0.05 vs. NPD-fed mice following recovery, ^#^*P* < 0.05 vs. NPD/HFHC-fed mice, ^§^*P* < 0.05 vs. LPD/HFHC-fed mice, Tukey’s *post hoc* test, One-way ANOVA).

To assess whether LPD followed by exposure to HFHC diet dysregulates glucose homeostasis, we assessed glucose tolerance and insulin resistance (Figs 3 and 4). We found that the absolute weight of pancreas as well as the ratio of pancreas weight to body weight in the LPD-fed mice were significantly lower than the control NPD-fed mice at the end of the LPD feeding period (Fig 3A and 3B). At recovery, although the absolute weight of pancreas or the pancreas-to-body weight ratio did not differ between NPD- or LPD-fed mice (Fig 3A and 3B), they were significantly higher in the LPD-fed mice compared to the LPD feeding period (Fig 3A and 3B). At the end of 16 weeks of HFHC feeding, the ratio of pancreas weight to body weight was significantly lower in the HFHC-fed mice compared to the chow diet-fed mice (Fig 3B), but the absolute pancreas weight was lower only in the LPD/HFHC-fed mice (Fig 3A). Furthermore, the pancreas-to-body weight ratio of the HFHC-fed mice was found to be significantly decreased after the 16 weeks of HFHC feeding compared to the end of the recovery period (Fig 3A).

The fasting blood glucose level was significantly higher only in the LPD/HFHC-fed mice at the end of 16 weeks of HFHC feeding as compared to chow diet-fed LPD mice (Fig 3C). At the end of LPD period, the LPD-fed mice had significantly lower fasting insulin compared to NPD-fed controls, and both the NPD and LPD groups following HFHC diet feeding had higher fasting insulin compared to the NPD and LPD groups fed chow diet (Fig 3D). At the end of LPD period, HOMA-IR was lower in LPD-fed mice compared to NPD-fed mice (Fig 3E). Following the HFHC diet feeding, both the NPD and LPD mice had higher HOMA-IR compared to the chow diet-fed mice (Fig 3E). We further assessed glucose tolerance by performing an IPGTT at the end of each diet period (Fig 4). We found no difference over time in their response to a bolus of glucose between NPD and LPD-fed mice at the end of LPD period (Fig 4A and 4B). However, the LPD-fed mice became glucose intolerant at the end of the recovery period when they were exposed to regular chow diet for 4 weeks (Fig 4C and 4D). Furthermore, we found that LPD/HFHC- and NPD/HFHC-fed mice had increased glucose area under the curve relative to NPD and LPD mice fed with chow diet for 16 weeks (Fig 4E and 4F). Overall, these findings indicate that during the recovery period the LPD-fed malnourished mice develop transient glucose intolerance but that the LPD feeding does not affect the level of glucose intolerance after HFHC diet in these mice.

### Impact of a nutritional double burden on hepatic features of NAFLD

**Fig 5:**
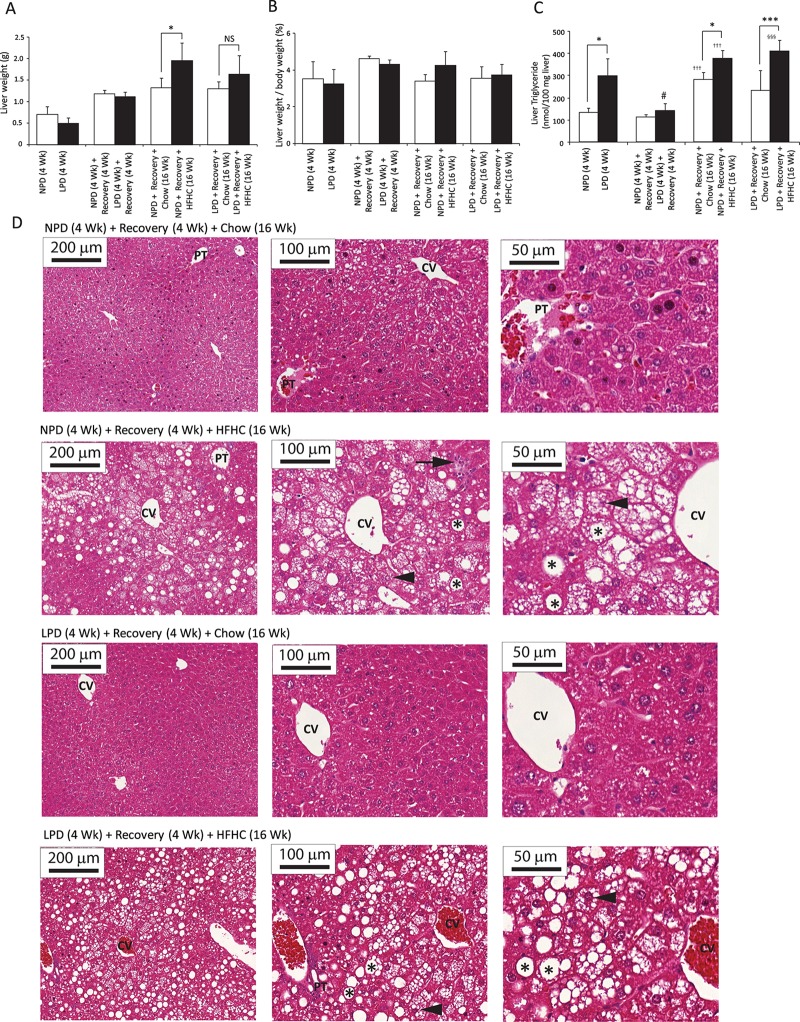
Assessment of liver pathology in C57BL/6 mice at the end of 4 weeks of normal protein diet (NPD, control diet) or low protein diet (LPD, malnutrition diet) feeding during the first burden/malnutrition period, 4 weeks of chow diet feeding during the recovery period, and 16 weeks of chow or HFHC diet feeding during the second burden period. (A) Mouse livers were weighed *ex vivo* after each feeding period. Only the NPD/HFHC-fed mice had greater liver weights than the chow diet-fed NPD mice (All data are presented as means ± SD, n ≥ 4 mice per group; *P < 0.05, NS (not significant) *P* value >0.05, Tukey’s *post hoc* test, One-way ANOVA). (B) Mouse livers were weighed ex vivo after each feeding period. The liver-to-body weight ratio did not differ between groups (All data are presented as means ± SD, n ≥ 4 mice per group; *P < 0.05, Tukey’s *post hoc* test, One-way ANOVA). (C) Liver triglycerides levels were determined at sacrifice. LPD-fed mice after 4 weeks had higher triglycerides level in liver than the NPD-fed mice, and the NPD/HFHC-fed as well as LPD/HFHC-fed mice had higher triglycerides levels than the corresponding chow diet-fed control groups. The liver triglyceride levels in the LPD-fed mice were significantly decreased post-recovery as compared with the malnutrition period (All data are presented as means ± SD, n ≥ 4 mice per group, *P < 0.05, ***P<0.001, ^†††^*P* < 0.001 vs. NPD-fed mice following recovery, ^§§§^*P* < 0.001 vs. LPD-fed mice following recovery, ^#^*P* < 0.05 vs. LPD-fed mice at the end of 4 weeks of malnutrition period, Tukey’s *post hoc* test, One-way ANOVA). (D) Histologic analysis. Hematoxylin-eosin staining of liver paraffin sections show normal histology in NPD-fed as well as LPD-fed mice following recovery and chow diet feeding for 16 weeks; inflammatory infiltrate (black arrow), and microvesicular (arrowhead) and macrovesicular (asterisk) steatosis were observed in NPD-fed and LPD-fed mice following recovery and HFHC diet feeding for 16 weeks; CV, central vein; PT, portal triad (magnification: X10, Bar = 200 μm; X20, Bar = 100 μm; X40, Bar = 50 μm).

To assess whether LPD feeding followed by HFHC diet feeding causes more severe hepatic steatosis and/or steatohepatitis, we evaluated key features of NAFLD/NASH in C57BL/6 mice. After the LPD feeding period, the liver absolute weights or the liver-to-body weight ratio did not differ between groups (Fig 5A and 5B). The levels of hepatic triglycerides in LPD-fed mice were significantly higher than in NPD-fed mice at the end of the 4 weeks of LPD period (Fig 5C). After recovery, the difference in liver triglycerides between NPD-fed and LPD-fed mice was no longer present (Fig 5C). After 16 weeks of HFHC feeding, the livers of HFHC-fed mice weighed more than those of chow diet-fed mice (Fig 5A); however, the ratio of liver weight to body weight did not differ in these mice (Fig 5B). The hepatic triglycerides were significantly higher in the HFHC-fed mice than in the chow diet-fed mice in both the NPD and LPD group (Fig 5C). Liver histology of NPD/HFHC-fed and LPD/HFHC-fed mice showed similar steatosis (Fig 5D, Table 3) and were significantly different from the corresponding chow diet-fed mice (Fig 5D, Table 3). In both the NPD/HFHC and LPD/HFHC groups, nearly all hepatocytes showed microvesicular steatosis, however, the macrovesicular steatosis was panlobular (Fig 5D). There were no overall differences in portal inflammatory features (Fig 5D) or fibrosis between groups as per the examination using Trichrome staining (data not shown). Distinct hepatocellular ballooning was not observed in HFHC diet-fed mice, however the hepatocellular hypertrophy was occasionally identified in mice fed with HFHC feeding (Fig 5D).

**Table 3:** Volume density of hepatic steatosis (Vv[steatosis, liver]) determined by image analysis method in liver sections stained with hematoxylin and eosin.

Data analysis	Vv[steatosis, liver] %			
	NPD (4 Wk) + Recovery (4 Wk) + Chow (16 Wk)	NPD (4 Wk) + Recovery (4 Wk) + HFHC (16 Wk)	LPD (4 Wk) + Recovery (4 Wk) + Chow (16 Wk)	LPD (4 Wk) + Recovery (4 Wk) + HFHC (16 Wk)
**Mean**	8.02	29.52**	9.61	25.06^§§^
**SD**	2.08	12.56	3.51	9.56
**SEM**	1.04	5.62	1.57	4.78

NPD: Normal protein diet; LPD: Low protein diet; HFHC: High fat high carbohydrate.

Data are expressed as the mean ± SEM (n ≥ 4 mice per group

**P<0.01 versus NPD-fed mice following recovery and chow diet feeding for 16 weeks

^§§^*P*<0.01 versus LPD-fed mice following recovery and chow diet feeding for 16 weeks, Tukey’s *post hoc* test, One-way ANOVA).

### Impact of a nutritional double burden on hepatic features of fibrogenesis, inflammation, oxidative stress and sucrose metabolism

**Fig 6:**
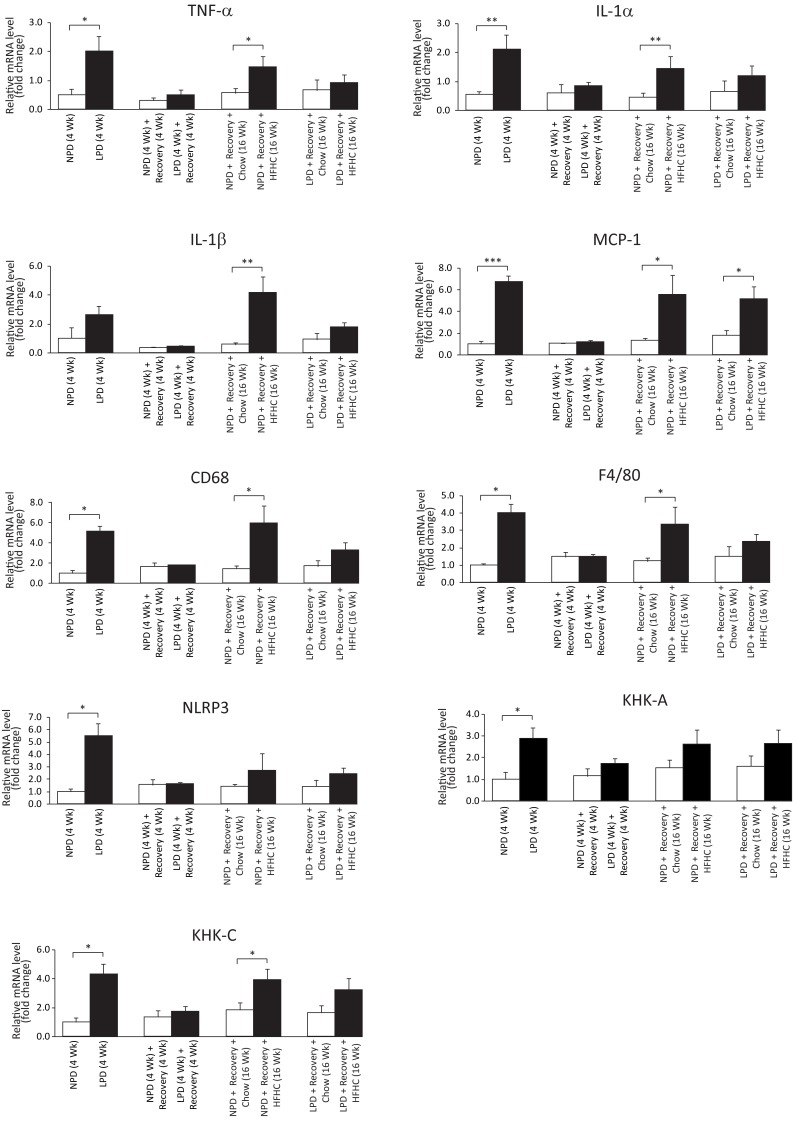
Assessment of liver gene expression in C57BL/6 mice at the end of 4 weeks of normal protein diet (NPD, control diet) or low protein diet (LPD, malnutrition diet) feeding during the first burden/malnutrition period, 4 weeks of chow diet feeding during the recovery period, and 16 weeks of chow or HFHC diet feeding during the second burden period. Hepatic mRNA levels of the proinflammatory cytokines (TNF-α, IL-1α, IL-1β, MCP-1), inflammasome component (NLRP3), and markers of inflammatory cells (CD68, F4/80) were measured by real-time RT-PCR and expressed in relative expression units to 18S ribosomal RNA (All data are presented as means ± SEM, n ≥ 4 mice per group, *P < 0.05, **P < 0.01, ***P < 0.001, Student's *t-*test).

**Fig 7:**
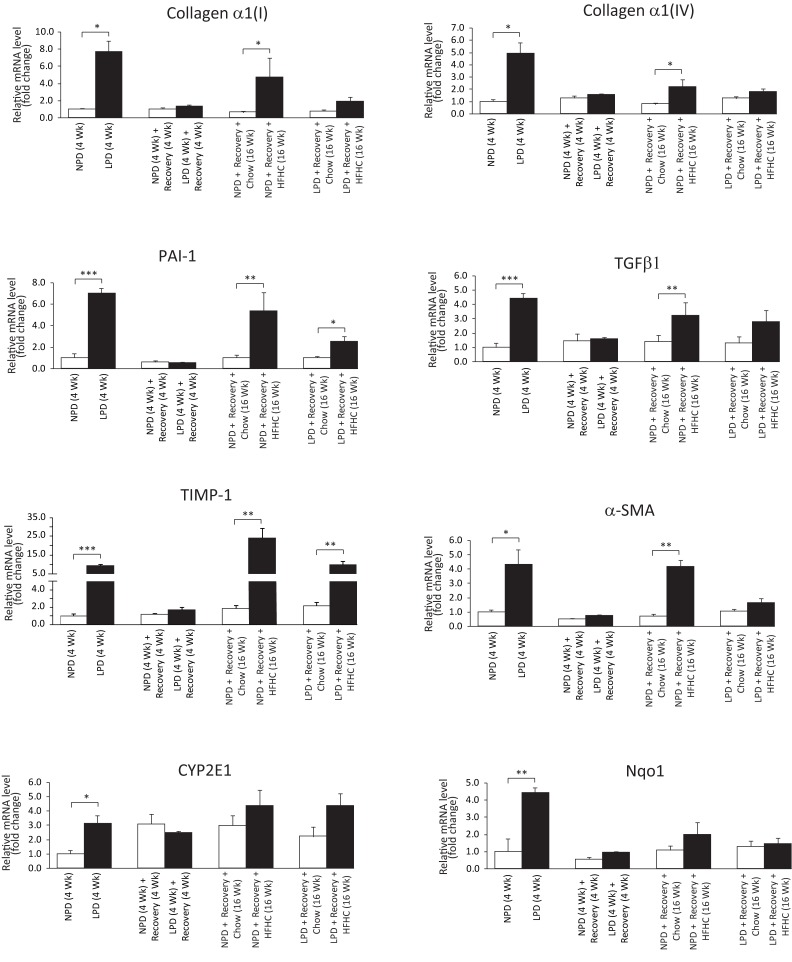
Assessment of liver gene expression in C57BL/6 mice at the end of 4 weeks of normal protein diet (NPD, control diet) or low protein diet (LPD, malnutrition diet) feeding during the first burden/malnutrition period, 4 weeks of chow diet feeding during the recovery period, and 16 weeks of chow or HFHC diet feeding during the second burden period. Hepatic mRNA levels of the profibrogenesis markers (Collagen α1(I), Collagen α1(IV), PAI-1, TGFβ1, TIMP1, α-SMA) and oxidative stress markers (CYP2E1, Nqo1) were measured by real-time RT-PCR and expressed in relative expression units to 18S ribosomal RNA (All data are presented as means ± SEM, n ≥ 4 mice per group, *P < 0.05, **P < 0.01, ***P < 0.001, Student's *t-*test).

To determine whether exposure to a double dietary burden impacts fibrogenesis, inflammation and oxidative stress in liver, we assessed liver histology, measured liver hydroxyproline content and investigated gene expression. Trichrome-stained liver sections did not show evidence for significant fibrosis in the liver (data not shown). Furthermore, the hydroxyproline content in the liver samples across the groups was not significantly different (S9 Data). However, a panel of profibrogenesis (Collagen α1(I), Collagen α1(IV), PAI-1, TGFβ1, TIMP1, α-SMA), inflammatory (TNF-α, IL-1α, MCP-1, CD68, F4/80, NLRP3) and oxidative stress marker (CYP2E1, Nqo1) genes were up-regulated after the LPD period (Figs 6 and 7). Similarly, after 16 weeks of HFHC feeding, all these genes, except CYP2E1, Nqo1 and NLRP3, were increased in the NPD/HFHC-fed mice (Figs 6 and 7). In contrast, only PAI-1, TIMP1 and MCP-1 were significantly upregulated in the LPD-fed mice exposed to the HFHC diet feeding (Figs 6 and 7). As sucrose intake is known to upregulate ketohexokinase (fructokinase) expression, we measured both isoforms of ketohexokinase (KHK-A and KHK-C). The principal isoform in the liver is KHK-C and is a rapid phosphorylator, and metabolizes fructose to fructose-1-phosphate rapidly, resulting in transient depletion of intracellular ATP that may have a role in inducing mitochondrial oxidative stress and fat accumulation [28]. An increase in the mRNA levels of both these isoforms at the end of LPD feeding period in the LPD-fed mice and an increase only in KHK-C expression was observed in the NPD-fed mice followed by HFHC feeding (Fig 6). Overall, most inflammatory and oxidative stress-related transcriptional changes occurred in the LPD-fed mice during the LPD feeding period. The relatively mild changes in the LPD-fed mice following HFHC diet feeding suggest that early exposure to low protein diet might be protective against liver fibrogenesis and inflammation in these HFHC-fed mice.

## Discussion

The prevalence of obesity and associated chronic disorders is rising in developing countries where malnutrition also prevails [1, 14, 29]. One potential long-term outcome for the survivors of acute malnutrition in infancy and early childhood is an increased risk of non-communicable diseases, such as type 2 diabetes and NAFLD [11, 30]. However, direct evidence for the relationship between acute malnutrition in infancy and development of NAFLD in adulthood is scarce. The present pre-clinical double-burden study was designed to understand the impact of early-life malnutrition through protein restriction on development of NAFLD and insulin resistance following an obesogenic diet in later-life in male mice. To our knowledge, this is the first study to assess the metabolic effect of post-natal protein restriction (first burden), recovery and later-life exposure to an obesogenic environment (second burden) in a mouse model.

Early-life malnutrition results in growth stunting [31]. In our early-life malnutrition model, slow growth rate was evident as the mice gained an average of 50% less weight after 4 weeks of protein restriction during the post-weaning period. At this time point, the malnourished mice were also stunted. With the recovery, these mice showed catch-up growth and were no longer stunted as long. We observed that with the obesogenic diet feeding these previously malnourished mice reached a lower weight compared with the NPD-fed mice.

In the present study, the energy expenditure did not differ between the LPD and the NPD-fed control mice during the initial period of LPD feeding, whereas at the end of recovery period, the LPD-fed mice demonstrated a decrease in energy expenditure during the light phase suggesting a metabolic response in favour of body mass conservation and gain during this catch-up growth period. A study conducted in recovering malnourished infants and age-matched healthy controls found that the recovering malnourished infants appeared to conserve energy through a reduction in physical activity, and increased sleep or inactivity [32]. At the end of obesogenic HFHC diet feeding, the energy expenditure in the NPD-fed control mice was significantly increased; however, the energy expenditure in the LPD-fed mice remained unchanged, most possibly, in favour of weight gain. The less energy expenditure could be due to a consequence of a lower increase in lean body mass, or other mechanisms, such as, decreased IGF-1 levels and a higher ratio of cortisol to insulin associated with malnutrition, acting in favour of conservation of energy and/or body fat [33].

The RER, an indicator of the relative rate of substrate oxidation, was significantly higher in the LPD-fed mice indicating increased whole-body metabolism with a greater dependence on carbohydrate as fuel during malnutrition. Similar to the LPD-fed mice in the present study, an elevated RER was also found in malnourished infants [32]. Furthermore, it may also reflect increased gluconeogenic activity to maintain energy supply to the brain [34]. After recovery, the LPD-fed mice exhibited decreased RER suggesting that these mice utilized a higher percentage of fat in their energy production than the matched control mice. However, the decreased RER in the LPD-fed mice was contrary to what was found in nutritionally stunted children, who demonstrated significantly higher respiratory quotients and measurably impaired fat oxidation compared with non-stunted control children living in the same environment [35]. The decreased RER during recovery and HFHC feeding found in the present study may reflect increased fat oxidation in mice during the recovery and obesogenic diet feeding periods in accordance with other studies using a similar diet in mice [36].

The developmental origins of health and disease (DOHaD) theory describes associations between *in utero* or very early postnatal insults, such as malnutrition, and adult non-communicable disease risk that includes development of obesity and type 2 diabetes [37, 38]. By applying the DOHaD theory, several epidemiological studies have related birth weight or size at birth to subsequent risk of developing insulin resistance and/or type 2 diabetes mellitus in middle age [37, 38]. Studies on famine events such as the Dutch Hunger Winter and the Great Chinese Famine have demonstrated that poor early-life nutrition can be associated with obesity, cardiovascular disease, and metabolic syndrome [39, 40]. However, very few studies have investigated the long-term implications of postnatal severe acute malnutrition. A recent follow-up study conducted in Malawian children aimed to explore the long-term effects of severe acute malnutrition [41]. This study observed that the survivors of early-life malnutrition showed persistent post-malnutrition stunting, and raised a possibility that it can be associated with future cardiovascular and metabolic disease [41]. In the present double-burden mouse study, we found that the stunting in the malnourished mice was reversible during the rapid catch-up growth when fed with regular chow diet during the recovery period, however, these mice developed glucose intolerance after initial recovery, as was observed by the IPGTT. However, insulin resistance developed similarly in both LPD-fed and NPD-fed mice after a HFHC diet, which means that either the secondary insult was so overwhelming it obscured the effect of earlier LPD feeding or that the glucose intolerance after LPD is transient.

The malnourished mice in our study developed transient liver steatosis as was found in previous studies that focused on postnatal malnutrition [17, 42]. However, the long-term risk of developing steatosis on a HFHC diet was equal in previously malnourished LPD-fed and control NPD-fed mice. There were minimal histological and biochemical signs of inflammation in our HFHC-fed animals. Several pro-inflammatory, profibrogenesis and oxidative stress marker genes were up-regulated only in the LPD-fed mice, however, after feeding HFHC diet, some of these pro-inflammatory genes were more upregulated only in the control NPD mice. One possible explanation is that the small differences in dietary patterns can influence certain host metabolic and inflammatory markers via microbial gene diversity and quantity [43]. The other possibility is that the early-life protein restriction may have terminated or resolved inflammation by an active compensatory process involving cytokines and other anti-inflammatory mediators [44]. Overall, our findings suggest that protein restriction in early-life induced immediate pro-inflammatory changes without clear long-term increased susceptibility for NASH.

NAFLD is highly associated with obesity, insulin resistance, and diabetes [45]. There are limited data that suggest that exposure to an unfavourable environment in early infancy may program NAFLD through direct effects on the liver and indirect effects via adiposity and metabolic dysfunction [46]. A recent epidemiological study suggested that exposure to famine in early-life can be associated with NAFLD consistent with the hypothesis that malnutrition in early-life may influence development of NAFLD in later-life [47]. Our results, however, do not support this hypothesis. One alternative explanation could be that the 4% protein diet that was used as malnutrition diet in our study was not severe enough to significantly affect the susceptibility to develop diet-induced NAFLD in later-life. Our regular chow and initial insult diets had a modestly higher fat content than the standard AIN-93M diet (4 gm% = 9.4 kcal%) which might have had some effect on our results. A recently published study from our group used a mouse model of postnatal malnutrition induced with a diet with 1% of calories from proteins [18], whereas, Brown *et al*. used a malnutrition-inducing diet that was moderately low in protein containing 7% calories from proteins [42]. The postnatal protein restriction regimen that we used was similar to a study previously conducted by us in rats that used a malnutrition diet containing 4% calories from proteins, and this particular study associated post-natal hepatic steatosis with severe mitochondrial and peroxisomal dysfunction [17].

High-fat high-fructose-fed C57BL6 mice have been shown to develop NASH with fibrosis at 16 weeks of HFHC diet feeding [22]. Although the control NPD-fed mice on HFHC diet developed signs of more extensive NAFLD with induction of inflammatory markers, they did not develop any noticeable histological changes representative of fibrosis or collagen deposition. One possible reason could be that there exists a window period for the exposure to HFHC diet that triggers development of NASH with fibrosis. Kohli *et al*. initiated the HFHC diet feeding in 8-week-old mice [22], whereas, we started the HFHC diet feeding when the mice were 11 weeks old. In addition, the mouse strain and origin of animals might have influenced the phenotype. There are several diet-induced models of NAFLD/NASH in small animals; however, consensus regarding the optimal model is lacking. Recent studies with the American lifestyle-induced obesity syndrome (ALIOS) diet using *ad libitum* solid or liquid high-fructose and high-trans fat diets in small animals successfully generated steatosis with inflammation, but without significant fibrosis or collagen deposition [48–50]. It remains possible that longer treatment with a HFHC diet in our study would have resulted in hepatic inflammation and other more robust signs of NASH.

In summary, we established a mouse model of early-life malnutrition. Utilizing this model, we were able to conduct a double-burden study for the first time to understand the impact of early-life malnutrition on the development of later-life NAFLD, glucose intolerance and insulin resistance in obesogenic environment. Our animal data suggest that a low protein diet in early-life does lead to a transient glucose intolerance after nutritional recovery but does not increase the susceptibility to diet-induced NAFLD and insulin resistance later in life.

## Supporting information

S1 Data Raw data Fig 1B, 1C and 1E. (XLSX)

S2 Data Raw data Figs 1D, 3A, 3B, 5A and 5B. (XLSX)

S3 Data Raw data Fig 2. (XLSX)

S4 Data Raw data Fig 3C and 3D. (XLSX)

S5 Data Raw data Fig 3E. (XLSX)

S6 Data Raw data Fig 4. (XLSX)

S7 Data Raw data Fig 5C. (XLSX)

S8 Data Raw data Table 3. (XLSX)

S9 Data Raw data for hydroxyproline assay. (XLSX)

S10 Data Raw data Fig 6. (XLS)

S11 Data Raw data Fig 6. (XLS)

S12 Data Raw data Fig 6. (XLS)

S13 Data Raw data Fig 6. (XLS)

S14 Data Raw data Fig 7. (XLS)

S15 Data Raw data Fig 7. (XLS)

S16 Data Raw data Fig 7. (XLS)
